# Supplementary material for: Magnesium supplementation alleviates drought damage during vegetative stage of soybean plants
Source: PLoS One. 2023 Nov 3;18(11):e0289018. doi: 10.1371/journal.pone.0289018 (PMC10624259; doi:10.1371/journal.pone.0289018)

Supporting information – S1 Fig

Article title: Magnesium supplementation alleviates drought damage during vegetative stage of soybean plants

Journal: Plos One

Author's names

Amanda Soares Santos[1], Davielson Silva Pinho[2], Alana Cavalcante da Silva[1], Ramilos Rodrigues de Brito[1], Julian Junio de Jesus Lacerda[1], Everaldo Moreira da Silva[1], Jennyfer Yara Nunes Batista[2], Bruno Sousa Figueiredo da Fonseca[2], Enéas Gomes-Filho[3], Stelamaris de Oliveira Paula-Marinho[1], Alexson Filgueiras Dutra[4], Marcos Renan Lima Leite[5], Alan Mario Zuffo[6], Francisco de Alcântara Neto[7], Jorge González Aguilera[8], José Antonio Rodríguez García[9], Pedro Arias Cubillas[10], Milko Raúl Rivera Campano[9], Alejandro Manuel Ecos Espino[9], Hebert Hernán Soto Gonzales[9], Rafael de Souza Miranda[1,*]

Affiliations

[1] Postgraduate Program in Agricultural Sciences, Federal University of Piauí, Bom Jesus, Piauí, Brazil;

[2] Agronomy Engineering Course, Federal University of Piauí, Bom Jesus, Piauí, Brazil;

[3] Postgraduate Program in Biochemistry, Federal University of Ceará, Fortaleza, Ceará, Brazil;

[4] Agronomy Engineering Course, Federal Institute of Piauí, Uruçuí, Piauí, Brazil

[5] Postgraduate Program in Agronomy, Federal University of Piauí, Teresina, Piauí, Brazil

[6] Department of Agronomy, State University of Maranhão, Balsas, Maranhão, Brazil

[7] Plant Science Department, Federal University of Piauí, Teresina, Piauí, Brazil

[8] State University of Mato Grosso do Sul, Cassilândia, Mato Grosso do Sul, Brazil;

[9] Universidad Nacional de Moquegua, Ilo, Peru;

[10] Escuela de Posgrado-Doctorado en Ciencias Ambientales, Universidad Nacional Jorge Basadre Grohmann, Tacna, Peru.

*** CONTACT**

Rafael de Souza Miranda
rsmiranda@ufpi.edu.br
Postgraduate Program in Agricultural Sciences, Campus Professora Cinobelina Elvas, Federal University of Piauí, Bom Jesus, PI, CEP 64900-000, Brazil;

S1 Fig. **Environmental data dynamics**. Dynamics of mean, maximum, and minimum temperature, relative humidity, and evapotranspiration after sowing.

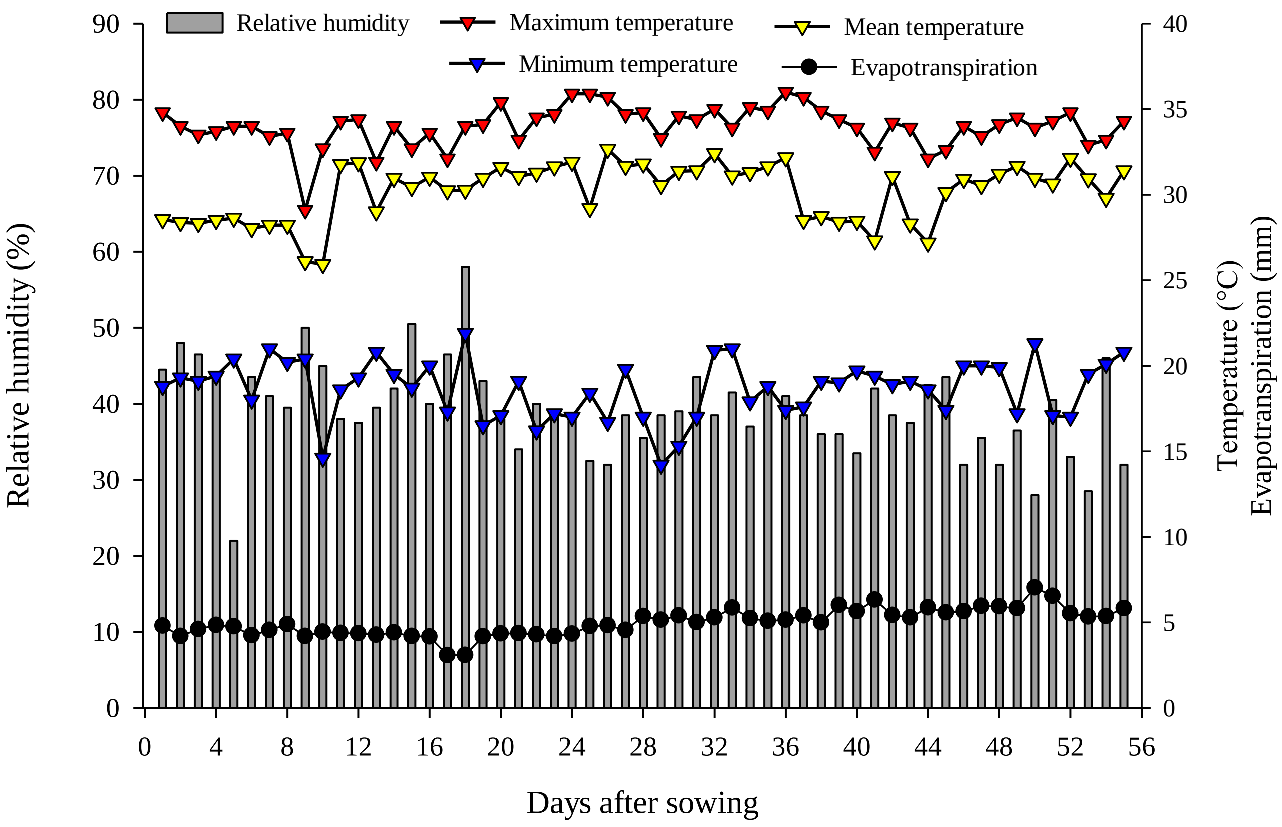

Supplement: S1 Fig — Dynamics of mean, maximum, minimum temperature, relative humidity and evapotranspiration after sowing. (PDF) [file pone.0289018.s001.pdf]
