# Supplementary material for: Magnesium supplementation alleviates drought damage during vegetative stage of soybean plants
Source: PLoS One. 2023 Nov 3;18(11):e0289018. doi: 10.1371/journal.pone.0289018 (PMC10624259; doi:10.1371/journal.pone.0289018)
Supplement: S3 Table — Split plot analysis of variance results for studied variables in soybean plants. (PDF) [file pone.0289018.s004.pdf]

Supporting information – S3 Table

Article title: Magnesium supplementation alleviates drought damage during vegetative stage of soybean plants

Journal: Plos One

Author's names
Amanda Soares Santos[1], Davielson Silva Pinho[2], Alana Cavalcante da Silva[1], Ramilos Rodrigues de Brito[1], Julian Junio de Jesus Lacerda[1], Everaldo Moreira da Silva[1], Jennyfer Yara Nunes Batista[2], Bruno Sousa Figueiredo da Fonseca[2], Enéas Gomes-Filho[3], Stelamaris de Oliveira Paula-Marinho[1], Alexson Filgueiras Dutra[4], Marcos Renan Lima Leite[5], Alan Mario Zuffo[6], Francisco de Alcântara Neto[7], Jorge González Aguilera[8], José Antonio Rodríguez García[9], Pedro Arias Cubillas[10], Milko Raúl Rivera Campano[9], Alejandro Manuel Ecos Espino[9], Hebert Hernán Soto Gonzales[9], Rafael de Souza Miranda[1,*]

Affiliations

[1] Postgraduate Program in Agricultural Sciences, Federal University of Piauí, Bom Jesus, Piauí, Brazil;

[2] Agronomy Engineering Course, Federal University of Piauí, Bom Jesus, Piauí, Brazil;

[3] Postgraduate Program in Biochemistry, Federal University of Ceará, Fortaleza, Ceará, Brazil;

[4] Agronomy Engineering Course, Federal Institute of Piauí, Uruçuí, Piauí, Brazil

[5] Postgraduate Program in Agronomy, Federal University of Piauí, Teresina, Piauí, Brazil

[6] Department of Agronomy, State University of Maranhão, Balsas, Maranhão, Brazil

[7] Plant Science Department, Federal University of Piauí, Teresina, Piauí, Brazil

[8] State University of Mato Grosso do Sul, Cassilândia, Mato Grosso do Sul, Brazil;

[9] Universidad Nacional de Moquegua, Ilo, Peru;

[10] Escuela de Posgrado-Doctorado en Ciencias Ambientales, Universidad Nacional Jorge Basadre Grohmann, Tacna, Peru.

*** CONTACT**
Rafael de Souza Miranda
rsmiranda@ufpi.edu.br
Postgraduate Program in Agricultural Sciences, Campus Professora Cinobelina Elvas, Federal University of Piauí, Bom Jesus, PI, CEP 64900-000, Brazil;

S3 Table. Summary of variance analysis (ANOVA) of studied variables. Split plot analysis of variance results for studied variables in soybean plants

	F value								
**Source of variation**	**PH**	**SD**	**NL**	**LA**	**SFM**	**SDM**	**TOL**	**Ψs**	**RWC**
Mg dose (D)	90.25**	28.62**	35.57**	31.33**	66.64**	44.18**	344.96**	2.61^{ns}	2.09^{ns}
Genotype (G)	57.55**	6.51**	0.95^{ns}	1.09^{ns}	3.51*	1.59^{ns}	7.39**	0.08^{ns}	46.02**
Water regime (WR)	99.20**	44.77**	60.83**	97.25**	129.41**	52.76**	77.42**	7.70**	24.18**
D × G	1.60 **	0.20^{ns}	2.28^{ns}	8.85**	5.41**	2.34^{ns}	21.45**	3.57*	4.70*
D × WR	13.53**	6.12**	2.18^{ns}	3.53*	2.17^{ns}	1.66^{ns}	8.74**	3.14^{ns}	3.26*
G × WR	14.50**	7.52**	3.39*	1.48^{ns}	0.77^{ns}	1.66^{ns}	2.26^{ns}	5.18**	1.53^{ns}
D × G × WR	3.66*	3.00*	4.64**	10.65**	7.91**	9.89**	5.64**	4.26*	2.83*
CV (%)	5.38	10.50	18.31	17.39	16.95	20.14	15.43	22.26	16.19
**Source of variation**	**LS**	**Chl *a***	**Chl *b***	**Chl *total***	**Car**	**Mg - leaf**	**Mg - stem**	**Chl *a*/*b* ratio**	**Car/Cho *total* ratio**
Mg dose (D)	12.70**	75.67**	94.27**	67.15**	47.61**	106.69**	99.17**	0.00^{ns}	0.04^{ns}
Genotype (G)	3.33*	4.23*	23.80**	5.54**	0.56*	2.21^{ns}	10.29**	15.93**	8.99**
Water regime (WR)	7.45**	136.39**	187.82**	134.29**	49.16^{ns}	64.15**	74.00**	11.11**	30.46**
D × G	0.30^{ns}	5.33**	33.56**	14.91**	0.12**	2.06^{ns}	5.60**	12.15**	42.76**
D × WR	0.13^{ns}	3.43*	10.43**	15.18**	9.82^{ns}	14.99**	9.46**	0.18^{ns}	0.38^{ns}
G × WR	2.62*	2.92*	7.59**	5.76**	3.96^{ns}	5.26**	4.42**	4.37**	2.73*
D × G × WR	6.69*	32.17**	57.87**	25.92**	19.98**	13.68**	19.23**	7.52**	26.34**
CV (%)	14.16	15.90	13.38	16.06	20.87	16.80	17.60	13.70	14.33

Note:
** significant at 1% probability, * significant at 5% probability, ^{ns} non-significant.
PH - plant height, SD - stem diameter, NL - number of leaves, LA - leaf area, SFM - shoot fresh mass, SDM - shoot dry mass, TOL - relative tolerance to drought, Ψs - osmotic potential, RWC - relative water content, LS - leaf succulence, Chl *a* - chlorophyll *a*, Chl *b* - chlorophyll *b*, Chl *total* - chlorophyll *total*, Car – carotenoids.
